# Psoriasis complicated with polymyositis successfully treated with Ixekizumab: A case report

**DOI:** 10.1097/MD.0000000000042550

**Published:** 2025-05-30

**Authors:** Shengli Chen, Jifeng Liu

**Affiliations:** a Department of Dermatology, Hangzhou Third People’s Hospital, Hangzhou, Zhejiang Province, China.

**Keywords:** Ixekizumab, polymyositis, psoriasis

## Abstract

**Rationale:**

Psoriasis is an immune-mediated chronic inflammatory skin disease that is rarely complicated by polymyositis in clinical practice. Here, we report a patient with psoriasis, following treatment with Etanercept, who exhibited an increase in creatine kinase (CK) levels and a decline in muscle strength. After combined treatment with methylprednisolone, methotrexate, and Ixekizumab, the psoriasis rash completely subsided and CK levels returned to normal.

**Patient concerns:**

The patient was a 22-year-old female with systemic erythema and scale itching for 3 months, and her serum CK level was 1711 U/L without muscle pain and muscle weakness. She was diagnosed with psoriasis and myositis awaiting investigation and was then treated with Etanercept at 50 mg weekly. At the 6-month follow-up with ongoing Etanercept treatment, her serum CK level was elevated to 3465 U/L. Electromyography and thigh magnetic resonance imaging revealed myositis. After methylprednisolone and methotrexate tablet treatment for polymyositis, she developed pulmonary pneumonia, and methotrexate and methylprednisolone tablets were withdrawn. Although the pneumonia was controlled, the facial erythema, plaques, scales, and rash gradually increased and spread all over the body. Moreover, gastrocnemius pain and fatigue persisted. The patient was treated with subcutaneous injection of 160 mg of Ixekizumab combined with methylprednisolone tablet (8 mg/d), after 2 weeks, the dosage was reduced to 80 mg once every 2 weeks, and the patient’s psoriastic rash had completely disappeared after 4 weeks. The patient continued to receive subcutaneous injections of Ixekizumab 80 mg once a month and methylprednisolone tablet 6 mg every day. Six months later, no rash recurrence was noted and her CK level was within the normal range.

**Diagnoses:**

The patient was diagnosed with psoriasis with polymyositis.

**Interventions:**

After combined treatment with methylprednisolone, methotrexate, and Ixekizumab, the psoriasis rash completely subsided and CK levels returned to normal.

**Outcomes:**

Our case shows that Ixekizumab is an effective treatment for psoriasis complicated with polymyositis, which is worth clinical application.

**Lessons:**

Although Etanercept can be used to treat psoriasis and dermatomyositis, it can also aggravate the myositis. Ixekizumab is an effective treatment for psoriasis complicated by polymyositis.

## 1. Introduction

Psoriasis is an immune-mediated chronic inflammatory skin disease with a prevalence of 2% to 3% worldwide, and it is rarely found to be clinically complicated with polymyositis. Psoriasis and polymyositis have similarities in that they share common cytokines involved in their pathogenesis, as well as abnormalities in T cell function; thus, they may have some commonalities in their pathogenesis. At present, biological agents such as tumor necrosis factor alpha (TNF-α) and interleukin-17 (IL-17A) inhibitors are widely used to treat psoriasis and have achieved good clinical efficacy. However, the efficacy of TNF-α inhibitors in the treatment of polymyositis is uncertain and has only been reported on a case-by-case basis, with no reports on IL-17A inhibitors in the treatment of polymyositis. Ixekizumab is an IgG4 monoclonal antibody that selectively targets IL-17A with high affinity, and it represents an effective and generally well-tolerated treatment for patients with moderate-to-severe plaque psoriasis.^[1,2]^ Ixekizumab has a rapid onset and is administered as a 4-weekly dosing regimen after a 3-month induction period. Research has shown that patients who do not respond to 12-week Etanercept treatment can achieve a good curative effect after a 12-week Ixekizumab treatment.^[3]^

Pustular psoriasis is a special type of psoriasis that is accompanied by fever, muscle pain, joint pain, and systemic inflammation. Although TNF-α inhibitors can be used to treat pustular psoriasis and dermatomyositis, they have also been reported to induce amyopathic dermatomyositis and polymyositis.^[4,5]^ Herein, we report a case of psoriasis in a patient following treatment with Etanercept, who exhibited an increase in creatine kinase (CK) and a decline in muscle strength. After treatment with Ixekizumab, the psoriasis rash completely subsided, and the CK level returned to normal.

## 2. Case report

**Figure 1.**
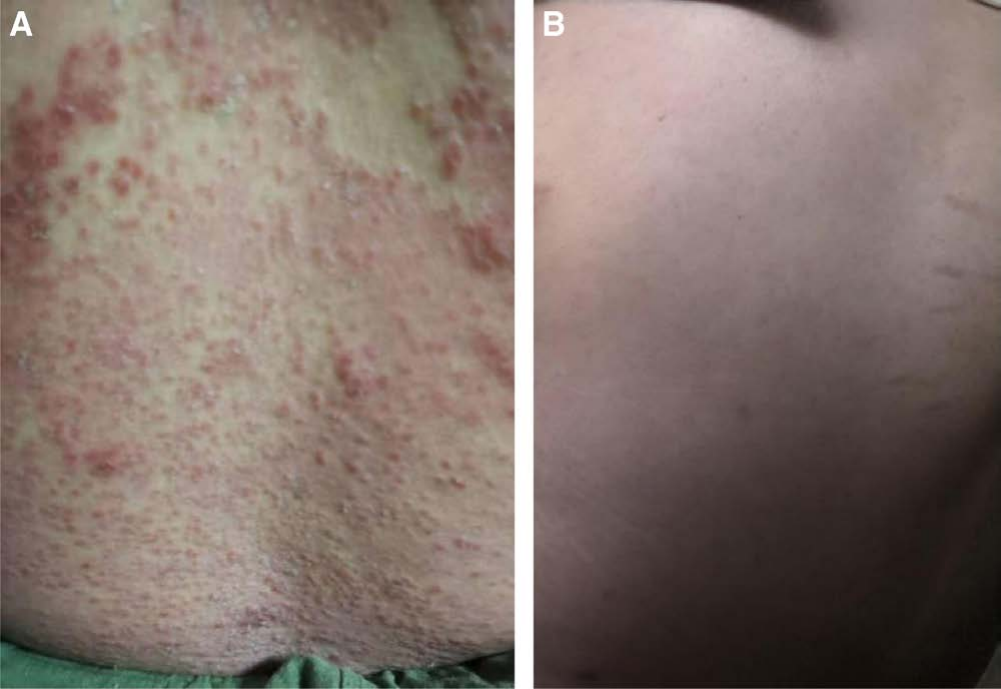
Rythematous, papules, pustules, and plaques of the lower limbs before treatment.

**Figure 2.**
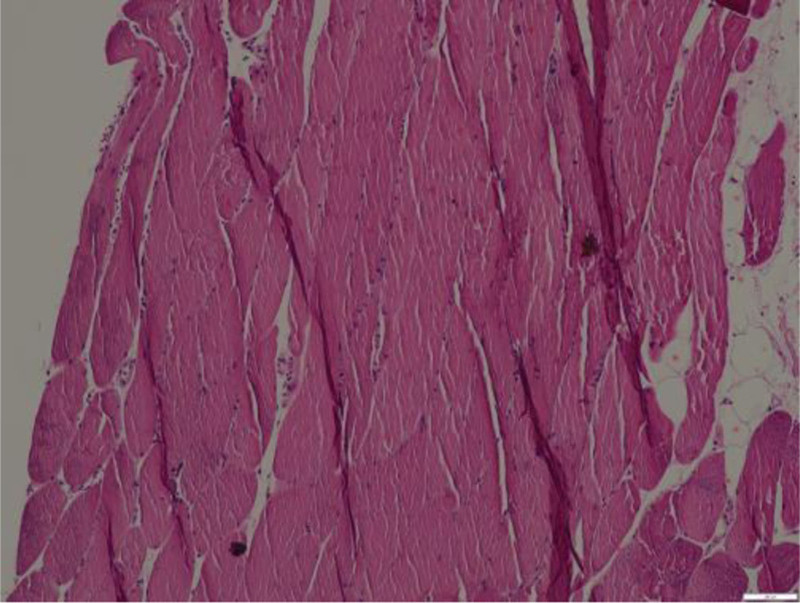
Mononuclear lymphocyte-like cell infiltration between muscle fibers.

**Figure 3.**
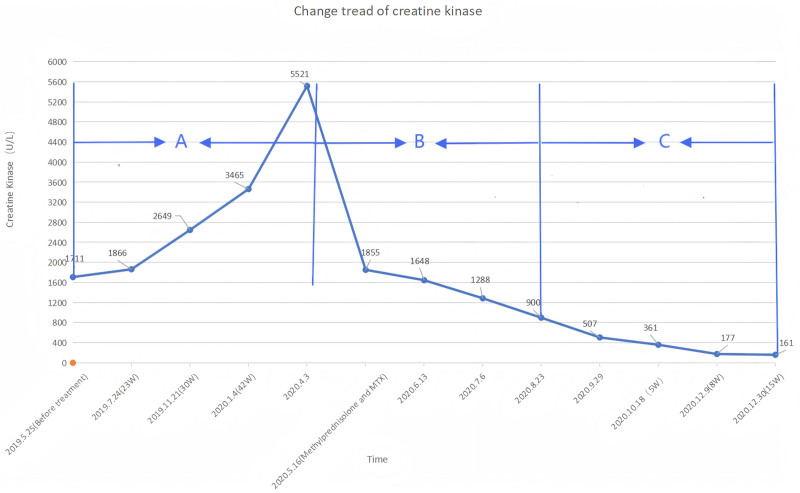
Chart showing the change in creatine kinase. (A) Treatment with Etanercept; (B) treatment with methylprednisolone and MTX; (C) treatment with Ixekizumab. MTX = methotrexate.

A 22-year-old female student presented to our hospital with systemic erythema and scale itching that had been present for 3 months; this had been accompanied by pustules for 3 weeks in May 2019. Upon initial examination, the patient was found to be obese, with a height of 160 cm and weight of 84 kg. The muscle strength of her 4 limbs was level 5 without tenderness, her temperature was 37.5°C, and she presented with generalized erythema and plaques of varying sizes over the skin of her whole body. These plaques were covered with dry scales, which were easy to scrape. The auspitz sign was positive. Additional pustules and lakes of pus were visible on the limbs (Fig. 1). Biochemical and imaging examinations revealed the following: CK, 1711 U/L; CK isozyme, 57 U/L; alanine aminotransferase, 120 U/L; aspartate aminotransferase, 105 U/L; C-reactive protein, 24.1 mg/L; lactate dehydrogenase, 351 U/L; and α-hydroxybutyrate dehydrogenase, 267 U/L. The antinuclear antibody and blood culture results were normal. Abdominal B-ultrasonography revealed the presence of a fatty liver. Based on her medical history and clinical manifestations, psoriasis was diagnosed. The patient was administered glycyrrhizin and a ceftriaxone sodium needle. After her body temperature returned to normal levels for 1 week and the pustules had completely subsided, treatment with Etanercept at 50 mg weekly was initiated. At the 6-month follow-up with ongoing Etanercept treatment, her rash had almost completely subsided; however, her serum CK level was elevated to 3465 U/L. Electromyography and thigh magnetic resonance imaging revealed changes in myositis, indicating the development of myositis. The patient experienced discomfort in the upper limb, thigh, and gastrocnemius muscle pain, chest tightness, and shortness of breath. Her CK level was 5521 U/L. Therefore, the patient was administered 50 mg of Etanercept and an intravenous injection of methylprednisolone at 60 mg once daily. Oral methotrexate tablets were continued at 7.5 mg/wk, before reducing to 24 mg of methylprednisolone tablets per oral dose, combined with 25 mg of methotrexate tablets per week. Due to the occurrence of pneumocystis pneumonia for 3 months after beginning treatment, the methotrexate tablets were withdrawn, and methylprednisolone tablets were gradually reduced to 10 mg/d. Although the pneumonia was controlled, the facial erythema, plaques, scales, and rash gradually increased and spread throughout the body, with obvious pruritus and no pustules. Moreover, gastrocnemius pain and fatigue persisted. Muscle biopsy revealed mononuclear lymphocyte-like cell infiltration between muscle fibers (Fig. 2). Furthermore, the idiopathic inflammatory myopathy spectrum suggested an anti-HMGCR IgG++. As a result, the patient was diagnosed with psoriasis with polymyositis and treated with subcutaneous injection of 160 mg of Ixekizumab combined with methylprednisolone tablet 8 mg/d, beginning in August 2020. After 2 weeks, the dose of Ixekizumab was reduced to 80 mg once every 2 weeks, and the patient’s psoriastic rash had completely disappeared after 4 weeks, leaving some discoloration. Her CK level reduced to 361 U/L and further to 161 U/L by December 2020. The patient continued to receive subcutaneous injections of Ixekizumab (80 mg) once a month and methylprednisolone tablets (6 mg) every day. Six months later, during follow-up, no rash recurrence was noted, and her CK level was within the normal range (Fig. 3).

## 3. Discussion

This case had a complicated course, in which the CK level significantly increased, and typical clinical and pathological manifestations of polymyositis appeared during the treatment of pustular psoriasis with Etanercept. Subsequently, pneumocystis pneumonia developed during treatment with methylprednisolone and methotrexate. The rash recurred during the course of reduction with methotrexate and methylprednisolone treatment. Finally, after treatment with Ixekizumab, the psoriasis rash subsided, the CK level returned to normal, and the PM symptoms disappeared.

Although psoriasis and polymyositis are different diseases, they share similar pathologies, involving abnormal T cell function and the production of TNF-α and IL-17. Moreover, the IL-17A level is associated with the disease activity of adult patients with dermatomyositis and polymyositis.^[6]^ Etanercept, a TNF-α inhibitor, was the 1st biologic agent to enter the clinic, and it provides an effective treatment for the majority of patients with moderate to severe psoriasis.^[7]^ Although Etanercept is also used to treat dermatomyositis, some reports have indicated that it can induce amyopathic dermatomyositis and polymyositis.^[4,5,8]^

The psoriasis rash in this patient subsided after treatment with Etanercept, but the muscle enzyme level increased significantly, and clinical and pathological manifestations of typical polymyositis appeared, further proving that Etanercept can aggravate myositis. In patients receiving Ixekizumab, the CK levels decreased to the normal range. Interestingly, Secukinumab, an IL-17 inhibitor, causes a disease flare of dermatomyositis.^[8]^ This difference may be related to the different pathogeneses of dermatomyositis and polymyositis, as well as the fact that Secukinumab is an IgG1 mAb targeting IL-17A, while Ixekizumab is an IgG4 mAb targeting IL-17A. To the best of our knowledge, this is the 1st case of successful treatment of psoriasis complicated by polymyositis. Our case study shows that Ixekizumab is an effective treatment for polymyositis, which is worthy of clinical application, following confirmation of our findings.

## Acknowledgments

We thank LetPub (www.letpub.com.cn) for linguistic assistance during the preparation of this manuscript.

## Author contributions

**Resources:** Shengli Chen, Jifeng Liu.

**Writing – original draft:** Shengli Chen.

**Writing – review & editing:** Jifeng Liu.
